# The effect of financial incentives on top of behavioral support on quit rates in tobacco smoking employees: study protocol of a cluster-randomized trial

**DOI:** 10.1186/s12889-016-3729-y

**Published:** 2016-10-06

**Authors:** F. A. van den Brand, G. E. Nagelhout, B. Winkens, S. M. A. A. Evers, D. Kotz, N. H. Chavannes, C. P. van Schayck

**Affiliations:** 1 Department of Family Medicine, Maastricht University (CAPHRI), P. Debyeplein 1, 6229 HA Maastricht, Netherlands; 2 Department of Health Promotion/Family Medicine, Maastricht University (CAPHRI), P. Debyeplein 1, 6229 HA Maastricht, Netherlands; 3 Department of Methodology and Statistics, Maastricht University, P. Debyeplein 1, 6229 HA Maastricht, Netherlands; 4 Department of Health Services Research, Maastricht University (CAPHRI), Duboisdomein 30, 6229 GT Maastricht, Netherlands; 5 Trimbos Institute, Netherlands Institute of Mental Health and Addiction, Centre for Economic Evaluations, Utrecht, Netherlands; 6 Department of Institute of General Practice, Heinrich-Heine University, P.O. Box 101007, 40001 Düsseldorf, Germany; 7 Department of Public Health and Primary Care, Leiden University Medical Center, Hippocratespad 21, 2333 ZD Leiden, Netherlands

**Keywords:** Smoking cessation, Incentives, Reward, Employees, Intervention, Tobacco

## Abstract

**Background:**

Stimulating successful tobacco cessation among employees has multiple benefits. Employees who quit tobacco are healthier, more productive, less absent from work, and longer employable than employees who continue to use tobacco. Despite the evidence for these benefits of tobacco cessation, a successful method to stimulate employees to quit tobacco is lacking. The aim of this study is to evaluate whether adding a financial incentive to behavioral support (compared with no additional incentive) is effective and cost-effective in increasing abstinence rates in tobacco smoking employees participating in a smoking cessation group training.

**Methods/design:**

In this cluster-randomized trial employees in the intervention and control group both participate in a smoking cessation group training consisting of seven weekly counseling sessions of ninety minutes each. In addition to the training, employees in the intervention group receive a voucher as an incentive for being abstinent from smoking at the end of the training (€50), after three months (€50), after six months (€50), and after one year (€200). The control group does not receive any incentive. The primary outcome is carbon monoxide validated 12-month continuous abstinence from smoking (Russel’s standard). Additionally, an economic evaluation is performed from a societal and an employer perspective.

**Discussion:**

The present paper describes the methods and design of this cluster-randomized trial in detail. We hypothesize that the financial incentive for abstinence in the form of vouchers increases abstinence rates over and above the group training. The results of this study can provide important recommendations for enhancement of employee tobacco cessation.

**Trial registration:**

Dutch Trial Register: NTR5657. First received 27-01-2016.

## Background

Tobacco use is a major health threat and responsible for deaths due to cancer, chronic obstructive pulmonary disease (COPD), coronary heart disease, stroke and heart failure [1]. The life expectancy for smokers is at least 10 years shorter than for nonsmokers [1, 2]. Not only is smoking a serious health risk, it is also responsible for an estimated 8.7 % of annual healthcare costs in the US [3]. Employees who smoke also represent a significant cost to their employers [4]. These costs can be distinguished as the cost due to sickness absenteeism and the cost due to smoking breaks. Smokers are about 1.5 more days per year absent from work than non-smokers [5, 6]. Smoking is associated with absenteeism [5, 7], and with reduced performance [8, 9]. The amount of time that employees spend smoking each workday depends on several factors, such as the number of smoking breaks and whether the employee has to go outside to smoke. An employee who works fulltime and takes four smoking breaks of ten minutes per day spends about 150 (work) hours per year on smoking. It was estimated that in the US, employees who smoke cost their employer an excess of $5816 per smoker per year [4]. On the other hand, when employees quit smoking, their absenteeism declines within several years [5]. In addition, the productivity of former smokers is higher than that of current smokers [5]. It is therefore profitable for employers to invest in smoking cessation both from a company perspective as well as from a societal viewpoint.

An approach in stimulating employees to stop smoking is to use incentives for quit success. The rationale behind incentives for healthy behavior is twofold. Firstly, people value present benefits and costs more than future ones [10]. Secondly, people are more motivated by tangible gains such as a financial benefit, than by long-term intangible gains like a reduction in the chance of negative health outcomes. These two irrational ‘decision biases’ can be used to nudge people toward healthy behavior that is beneficial for them in the long run [11]. An incentive in the form of money or vouchers can be the immediate, concrete reward which can motivate people to stop smoking.

Several studies have shown that financial incentives for smoking cessation success can be an effective method to stimulate smoking cessation [12–18]. For instance, incentives have proven to be successful in increasing continuous abstinence in pregnant women [15], in homeless smokers [16], and in substance abusers [17]. A long-term study by Volpp et al. [18] conducted in the United States involved 878 participating employees of a multinational corporation. Participants were given the opportunity to follow behavioral counseling for smoking cessation near their hometown and, if desired, in combination with pharmacological treatment. Both behavioral counseling and medication were fully reimbursed by the employer. Participants in the intervention group received an incentive of $100 for completing a smoking cessation program, $250 for smoking abstinence at 6 months after study enrollment, and $400 for smoking abstinence at 12 months after study enrollment. Participants in the intervention group (with incentive) were significantly more likely to be completely abstinent after 6, 12, and 15 months than in the control group. Furthermore, participants in the intervention group were more likely to have started a smoking cessation program and to have completed the program. While incentives thus can motivate smokers to enroll in a cessation program, only few participants in the Volpp study [18] actually did enroll (incentive group 15.4 % vs control group 5.4 %). Importantly, the rate of quitters was substantially higher in participants from the incentive group who participated in the smoking cessation program than participants from the control group who enrolled in the training (46.3 % vs 20.8 %). It is therefore likely that the combination of an effective smoking cessation program with incentives for quit success will prove to be the most effective in increasing smoking cessation rates.

The chance to quit successfully increases when people receive professional stop smoking support [19]. Yet, smoking cessation treatment is relatively expensive for people with low incomes and the cost of medication can be a barrier [20]. Since a lower income is also related to a higher smoking prevalence [21], it is particularly important to make smoking cessation counseling accessible for people with lower incomes. If smoking cessation therapy is fully reimbursed, more smokers will make use of it which can lead to twice as many long term quitters [22, 23]. It is therefore conceivable that smokers are more willing to start a smoking cessation treatment when their employer accounts for the costs. For a smoking cessation training combined with incentives to be widely adopted by commercial companies as a common smoking cessation intervention, it needs to be cost-effective. To our knowledge, no study that has investigated the effect of incentives on long-term smoking abstinence in a workplace setting has incorporated an economic evaluation. We are only aware of a randomized controlled trial in pregnant women which found that incentives of 400 pounds were highly cost-effective [24]. Smoking cessation training in combination with incentives might therefore be attractive to employers in light of employee health improvement but also from a cost saving perspective.

The proposed study: “Continuous Abstinence Through Corporate Healthcare” (CATCH) is conducted in Dutch companies. Currently, about a quarter of Dutch adults smoke. Smoking prevalence is much higher among low (28 %) and moderate educated adults (27 %) than among high educated adults (19 %) [25]. This socioeconomic difference has been increasing over time in the Netherlands [26]. The higher prevalence of smoking in individuals from lower socioeconomic groups is the single most important cause of socioeconomic differences in mortality [27, 28]. In order to decrease these differences, it is vital to specifically target the lower socioeconomic population.

The aim of this study is to investigate whether financial incentives combined with a smoking cessation training can improve quit success. In addition, an economic evaluation is conducted to assess the costs and benefits of this intervention. The results of this study can provide key evidence for the applicability and effectiveness of incentives for smoking cessation and can offer recommendations for implementing incentives to reduce employee smoking in a corporate context.

## Methods/design

The primary aim of this study is to evaluate whether adding a financial incentive (compared with no additional incentive) is effective and cost-effective in increasing 12-month continuous abstinence rates in tobacco smoking employees participating in a smoking cessation group training. A secondary aim is to investigate the effect of the incentive on the quit rate immediately after the training, after three and after six months. Furthermore, this study investigates the cost-effectiveness of the incentive in terms of quit rate and utilities, both from a societal perspective and from the employer’s perspective.

**Fig. 1:**
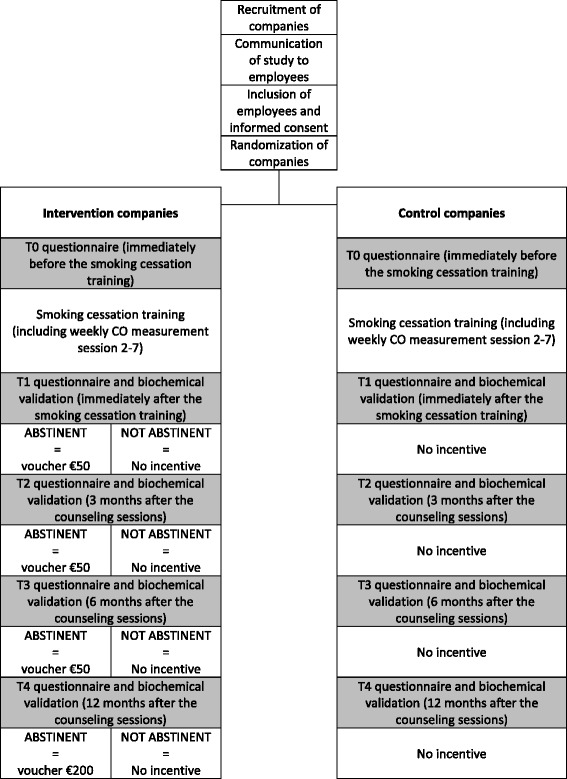
Flow-chart of design and measurements

A cluster-randomized controlled trial is conducted in 44 Dutch companies who offer their employees an evidence-based treatment for smoking cessation. Participants from the intervention companies receive incentives when smoking abstinence is achieved at a fixed time schedule (Fig. 1), whereas participants from the control companies do not receive incentives (care as usual).

### Study population

Approximately 516 tobacco smoking employees, randomized from 22 intervention companies and 22 control companies, participate in this study. To be eligible for participating in the study, companies have to meet certain criteria: (1) the management is willing to pay for the counseling sessions and (2) the management agrees that employees participate in the counseling sessions and carbon monoxide (CO) measurements during working hours or directly after on a location arranged by the company. In order to be eligible to participate in this study, an employee needs to be a current tobacco smoker and at least 18 years old. At the request of the employer, spouses who smoke are allowed to enroll in the study. Subjects are not eligible for participation in this study when they have an acute life-threatening disease, are not able to read or speak the Dutch language, or have already started an attempt to quit smoking at the moment of inclusion.

### Recruitment

In order to recruit companies, SineFuma - an organization that provides smoking cessation group training to companies - presents their clients with the possibility of participating in this research project. Additionally, the research assistant actively approaches companies by telephone and via a recruitment letter sent by post or e-mail with a visually attractive flyer attached. Press releases, media interviews and social media (Facebook, LinkedIn, Twitter) are used to create awareness of the study, and advertisements are placed in digital newsletters of several organizations, such as the Dutch Cancer Society and Lung Alliance Netherlands. Companies are targeted based on number of employees (n > 200) and socioeconomic status of employees (preferably lower educated workers).

In the recruitment process, companies are informed that they can be randomized to the incentive group or the control group. To which group the company is assigned is revealed during the first session of the smoking cessation training. This means that a company needs to decide to participate in the research without knowing the final result of the randomization.

When a company decides to participate in the study, participants within the company are recruited using advertisements via the corporate website, email, posters and informative flyers that are spread through the company building. On these promotion materials it is advertised that participants have a chance to earn financial incentives with a total amount of 350 euro. On request of the company, SineFuma or the research assistant organizes an informal presentation at the company to inform employees who consider participating in the training. Prior to enrolling in the study, employees receive an information letter and are asked to give their signed informed consent. In this information letter, the chance of being eligible to earn the financial incentives is explained, and detailed information on the smoking cessation training and the research procedures is provided.

### Intervention

#### Smoking cessation counseling

Evidence-based smoking cessation counseling is provided by experienced trainers of SineFuma to both employees in the intervention companies and the control companies. Groups of approximately 8–16 participants receive counseling in seven sessions of ninety minutes on a location arranged by the company. The training is practical in nature and focuses on ways to quit successfully and how to avoid relapse. It relies on a buddy system group dynamic to effectively promote peer support. The trainer informs participants about the possibility to use smoking cessation aids like nicotine replacement therapy and medication during their quit attempt. The company decides whether it reimburses the medication or if their employees have to claim it from their health insurance. After two sessions in which the participants are prepared, they stop smoking at the third session. In session four to seven, the participants receive counseling to get through the first difficult weeks of smoking abstinence. As a standard part of the smoking cessation training, participants perform a CO measurement every week starting the second session, which serves as a tool to motivate and encourage them to stay quit.

#### Incentives

As part of the intervention, participants from the intervention group who are continuously abstinent from smoking receive financial incentives. There is a fixed time schedule for the incentives to help maximize the effect on smoking cessation success. At T1, T2, and T3, participants who report to have been abstinent since the end of the smoking cessation training (continuous abstinence) and who have a CO score lower than 10 ppm [29] at the respective visit receive €50 credit. At T4, abstinent participants are given credit representing a value of €200. Participants who did not succeed in quitting smoking at T1 get a second chance to earn a voucher at T2 and to be eligible for the vouchers at T3 and T4 if they are verifiable smoking abstinent at T2 (point prevalence abstinence). The participants receive a digital code by e-mail which they can use to access a digital gift shop where they can exchange their voucher for a broad range of products and activities.

### Outcomes

The primary outcome is carbon monoxide validated 12 months (T1-T4) continuous abstinence from smoking (Russel’s standard) [29]. This is evaluated by self-report of smoking and validated by biochemical measurements. Self-report of smoking abstinence is assessed by measuring seven-day point prevalence abstinence and prolonged abstinence [29] and biochemically verified using a CO measurement. Abstinence is defined as smoking not more than five cigarettes from the start of the abstinence period. Secondary outcomes are smoking abstinence immediately after smoking cessation counseling (T1), after three months (T2) and after six months (T3). The same methods and definitions to determine smoking abstinence are used as for the primary outcome.

#### Carbon monoxide measurement

Smoking abstinence is biochemically validated by measuring expired air carbon monoxide concentrations using a cut-off point of 9 parts per million among those who report having quit smoking using a handheld monitor (PiCO™ Smokerlyzer, Bedfont Scientific Ltd., Kent, England). This is a non-invasive and reliable method to detect recent smoking [29]. As a part of the smoking cessation training, participants will perform a CO measurement in session 2–7. After the training, CO measurements will be performed by the research assistant to validate smoking abstinence. The validation takes place at the company within two weeks after distribution of the questionnaire at T1, T2, T3, and T4. When there is discordance between self-report of smoking abstinence and biochemical validation or when participants do not cooperate with biochemical validation, participants are assumed to be smokers [29].

#### Questionnaires

Participants fill out web-based questionnaires about their smoking and cessation behavior, use of resources (health care and other societal costs), and generic quality of life at T0 - T4. At T0, participants are asked about demographic characteristics, current and past smoking (cessation) behavior, number of pack years smoked [30], quit intention, use of smoking cessation treatment, and nicotine dependence [31]. At T1, participants are asked to evaluate the counseling sessions via multiple choice questions concerning the quality of the training and the coach, the individual components of the training, and the fact that the training was organized within the company. At T1 - T4, self-report of smoking abstinence is assessed by measuring seven-day point prevalence abstinence and prolonged abstinence [29]. At T4, participants from the intervention group are asked to evaluate the incentives via five multiple choice questions about appreciation of the incentives and their perceived effectiveness. At all measurement points, participants are asked about quit intention, use of smoking cessation treatment, nicotine dependence, stress, attitudes, self-efficacy, risk perception, (peer) social support, smoking regulations at the workplace, medical care and medication use.

#### Process evaluation

A process evaluation, consisting of questionnaires and interviews, is conducted to assess participants’ experience with the study. At 6 months after the smoking cessation training, qualitative interviews are conducted with participants from the intervention companies who quit smoking successfully and participants from the intervention companies who did not. At least 15 interviews are conducted, after which the point of data saturation will be reached if in three further consecutive interviews no new themes or relationships between themes have emerged [32]. From the participants who did not manage to quit, participants are interviewed who did not accomplish smoking abstinence in the early phase during the training, and participants who relapsed later on. The aim of the interviews is to gain insight in the participants’ motivation to enroll in the training, to hear their opinion on the effect of the incentive and to inquire their appraisal of the program. The interviews are recorded, transcribed and coded using NVivo software. Coding is performed by two independent researchers. Discrepancies are discussed with a third researcher. Researchers involved in interviewing and data analysis keep a diary to evaluate their own subjective views on the interpretation of the data.

### Sample size calculation

The study from Volpp et al. [18] is used to obtain estimates for the sample size calculation. After 12 months, 15 % of participants in the intervention group and 5 % in the control group were abstinent. With an alpha of 0.05, the required sample size to obtain this clinically relevant effect size with 80 % power is 141 participants per treatment group, based on the Chi-square test.

Since the randomization is on cluster (company) level and assuming a mean number of participants per company (m) of 12 and an intra-class correlation (ICC) of 0.05 [31], the design effect 1 + (m-1) ICC = 1.55, yields a sample size of 219 participants per group. Taking into account 15 % loss to follow up due to unexpected employee turnover [18], 516 participants (44 companies) have to be included in the study in total.

### Randomization

Participants are allocated via cluster-randomization on a company level to the intervention or control group. For large corporations with several branches in which the participants are not in direct contact with each other, each location is entered separately in the randomization. The randomization procedure is performed by an independent research assistant. The sequence of the randomization is generated with a digital randomization program using the biased urn method [33, 34], where the proportion of being randomized into a group is inversely related with the proportion of participants who are already randomised into that group, in order to maintain treatment balance throughout the trial.

### Statistical analysis

Based on the intention-to-treat principle, all randomized participants are included in the denominator for calculating abstinence rates with the exception of unavoidable loss to follow-up as stated the Russel Standard [29]. The primary effectiveness analysis examines the difference in prolonged smoking abstinence between intervention group and control group over a period of twelve months after the counseling sessions. To be able to account for repeated measures and nesting of participants within companies, generalized mixed models with the logit link are used. Time, group (intervention or control) and the interaction between time and group are entered in the model as fixed factors. Baseline measurements such as socioeconomic status (based on income and education) and nicotine dependence are considered as potential effect modifiers, and will therefore be explored by moderation in the analysis. If those are indeed effect-modifiers, the effects for each level of socioeconomic status and nicotine dependence are reported. In case of missing values in these potential effect modifiers, multiple imputation approach is used. Missing data in the outcome variable are not being imputed, since the likelihood-based approach is used to deal with missing values [35]. A two-tailed test is considered statistically significant with p-values <0.05.

### Economic evaluation

An economic evaluation in the form of a cost-effectiveness (CEA) and cost-utility analysis (CUA) from a societal perspective and from an employer’s perspective [36] is embedded in this trial-based economic evaluation. The time horizon and the measurement point are combined with the effectiveness study, i.e. the T0-T4 measurement. The primary outcome for the CEA is cost per continuously abstinent ex-smoker. The primary outcome of the CUA is cost per Quality Adjusted Life Year (QALYs). The utility value derived from the standard quality of life questionnaire, EuroQol 5D5L [37], using Dutch tariff [38] will be used to compute QALYs. The utilities at the various time points are used to compute QALYs by means of the area under the curve method [39].

As this economic evaluation is also performed from a societal perspective, this implies that all relevant costs and outcomes are taken into account. A separate analysis is performed from the employer’s perspective. It is hypothesized that the intervention is associated with an increased number of quitters, increase in quality of life, and decreased costs. The time horizon is 12 months. Costs (the use of resources) are measured continuously; outcomes for the economic evaluation study are measured before the start of the smoking cessation training, at 3 months, 6 months, and 12 months.

#### Cost measurement

Total costs are estimated using a bottom-up (or micro-costing) approach, where information on each element of service used is multiplied by an appropriate unit cost and summed to provide an overall total cost. Intervention costs, healthcare costs, respondent and family costs, and costs outside the health care sector are assessed, especially the cost for the employer. A cost questionnaire was especially designed for this study, based on existing questionnaires [40–43], which identifies all relevant costs aspects. Subjects are asked to report the data from their cost questionnaire relating to the previous 3 months at T0, and relating to the period in between measurements at T1-T4. The valuation of costs is based mainly on the updated Dutch manual for cost analysis [44]. Cost prices are expressed in 2017 euros. If necessary, existing cost-prices are updated to 2017 using the consumer price index.

#### Analysis of economic evaluation

The analysis of the economic evaluation contains several uncertainty analyses, including bootstrapping analysis for sample uncertainty. The results of the economic evaluation are presented in cost-effectiveness plane and cost-effectiveness acceptability curves (CEAC). The Incremental cost-effectiveness ratio (ICER) is determined on the basis of incremental costs and effects of evidence-based interventions for smoking cessation in a corporate setting compared to care as usual. The cost-effectiveness ratio is stated in terms of costs per outcome rate, the cost-utility ratio focuses on the net cost per Quality Adjusted Life Year gained.

The ICER is calculated as follows. ICER = (Ci – Cc)/(Ei – Ec), where Ci is the annual total cost of the evidence-based interventions for smoking cessation in a company setting group, Cc is the annual total cost of the care as usual group, Ei is the effects at one year follow-up for the evidence-based interventions for smoking cessation in a company setting group and Ec is the effect at one year follow-up for the care as usual group.

The robustness of the ICER is checked by non-parametric bootstrapping. Bootstrap simulations are also conducted in order to quantify the uncertainty around the ICER, yielding information about the joint distribution of cost and effect differences. The bootstrapped cost-effectiveness ratios are subsequently plotted in a cost-effectiveness plane, in which the vertical line reflects the difference in costs and the horizontal line reflects the difference in effectiveness. The choice of treatment depends on the maximum amount of money that society is prepared to pay for a gain in effectiveness, which is called the ceiling ratio. Therefore, the bootstrapped ICERs are also depicted in a cost-effectiveness acceptability curve showing the probability that evidence-based interventions for smoking cessation in a business setting is cost-effective using a range of ceiling ratios. Additionally, to demonstrate the robustness of our base-case findings, a multi-way sensitivity analysis is performed. In the sensitivity analysis, uncertain factors of assumptions in the base case analysis are recalculated in order to assess whether the assumptions have influenced the incremental cost-effectiveness ratio (ICER), for example by varying cost-prices and volumes between minimum and maximum [45].

## Discussion

This paper presents the protocol of the intervention study “Continuous Abstinence Through Corporate Healthcare” (CATCH). The aim of the study is to investigate the effectiveness of incentives on continuous abstinence in employees. A total of 516 employees, divided over 22 control and 22 intervention companies participate in a cluster-randomized controlled trial. In both groups, participants follow a seven-week smoking cessation program. In the intervention group, employees additionally receive vouchers for cessation success up to a total value of 350 euro. It is hypothesized that employees in the incentive group achieve a higher 12-month continuous abstinence rate.

### Strengths and limitations

The proposed study has several strengths, starting with its company setting. A company setting can have several advantages compared to other settings; corporations have the potential to reach a large amount of people, can motivate their employees to participate and thereby achieve high attendance rates, and may inspire peer support and positive peer pressure among colleagues [46]. Moreover, businesses have an interest in keeping their employees healthy [4].

Another strong point of this study is that the proposed study performs a cost-effectiveness and cost-utility analysis from a societal perspective and specifically from an employer’s perspective. To our best knowledge, the cost-effectiveness of financial incentives to stimulate long term smoking cessation in a company setting has not been established yet. The outcomes of the cost-effectiveness analysis can help inform employers who are considering providing smoking cessation support in combination with incentives for their employees.

What makes this study also particularly relevant is its target population of employees with a lower socioeconomic status. People with a lower education and income smoke more often [25, 47], smoke more cigarettes per day and have lower quit rates than people with a higher SES [26, 48]. Although smokers with low SES are as likely to start a quit attempt as smokers with a high SES, they are less likely to succeed in quitting smoking [49]. Potential barriers in quitting successfully are suggested to be lack of social support and financial barriers to smoking cessation treatment [47]. The current intervention aims to remove potential obstacles and to make the smoking cessation program easily accessible for employees in three ways: (1) by providing it via the employer, (2) by organizing the training sessions at the workplace, and (3) by designing the training as a group program so that colleagues can support each other both during and between sessions.

A possible limitation of this study is the lack of blinding of the research participants to the existence of the intervention and control conditions. It is expected that the incentive group is more attractive to subjects than the control group. If smokers would know for certain that the incentive is within reach, it could lead to employees signing up who only smoke occasionally and are not dependent on the training for a successful quit attempt. In contrast, the control group may appeal only to heavy smokers who are highly motivated to quit. If nothing would be arranged to avoid these potential problems, it was anticipated that the study would end up with a substantial larger number of subjects in the intervention condition than in the control condition, which could cause selection bias. To avoid this possibility, it was decided not to randomize participants prior to enrolment, but to inform them merely about their 50 % chance of being eligible to earn the vouchers. Only after enrolment, during the first training session, it is revealed whether participants are randomized into the control or intervention condition. This approach could, however, lead to attrition bias, when participants in the control group are disappointed about not being able to earn the vouchers and therefore drop out more frequently. Furthermore, it is anticipated that some larger companies will participate with more than one group. In order to avoid inequality among coworkers, it was decided that all groups within the same location of the corporation would be randomized together. A disadvantage of this decision is that when these groups do not start at the same time, it could cause selection bias, since employees in the second group of the company may find out whether they will receive the incentive or not.

## Conclusion

The proposed study is the first in the Netherlands that investigates the effect of financial rewards on smoking cessation and the first study worldwide that assesses the cost-effectiveness of incentives to decrease smoking in employees. The results of this study can provide important recommendations for the use of financial incentives to motivate employees to quit smoking.

## Abbreviations

CATCH: Continuous Abstinence Through Corporate Healthcare; title of the project
Cc: The annual total cost of the care as usual group
CEAC: Cost-effectiveness acceptability curves
Ci: Annual total cost of the evidence-based interventions for smoking cessation in a company setting group
CO: Carbon monoxide
COPD: Chronic obstructive pulmonary disease
Ec: The effect at one year follow-up for the care as usual group
Ei: The effects at one year follow-up for the evidence-based interventions for smoking cessation in a company setting group
ICER: Incremental cost-effectiveness ratio
